# Shelf-Life Optimisation of Plasma Polymerised (2,2,6,6-Tetramethylpiperidin-1-yl)oxyl (TEMPOpp) Coatings; A New Possible Approach to Tackle Infections in Chronic Wounds

**DOI:** 10.3390/antibiotics10040362

**Published:** 2021-03-29

**Authors:** Kilian Böttle, Krasimir Vasilev, Thomas Danny Michl

**Affiliations:** Future Industries Institute, University of South Australia, Mawson Lakes, SA 5095, Australia; kili.boettle@googlemail.com (K.B.); krasimir.vasilev@unisa.edu.au (K.V.)

**Keywords:** TEMPO, plasma polymer, chronic wound, shelf-life, XTT, EPR, S. *aureus*, S. *epidermis*, nitroxides, long-lived radical

## Abstract

Chronic wounds fail to heal and are accompanied by an ongoing infection. They cause suffering, shorten lifespans, and their prevalence is increasing. Unfortunately, the medical treatment of chronic wounds has remained unchanged for decades. A novel approach to break the biological vicious cycle is the long-lived radical (2,2,6,6-Tetramethylpiperidin-1-yl)oxyl (TEMPO). TEMPO can be plasma polymerised (TEMPOpp) into thin coatings that have antimicrobial properties. However, due to its radical nature, quenching causes it to lose effectiveness over time. Our aim in this study was to extend the shelf-life of TEMPOpp coatings using various storage conditions: Namely, room temperature (RT), room temperature & vacuum sealed (RTV), freezer temperature & vacuum sealed (FTV). We have analysed the coatings’ quality via the surface analytical methods of X-Ray Photoelectron spectroscopy (XPS) and electron paramagnetic resonance (EPR); finding marked differences among the three storage conditions. Furthermore, we have compared the antimicrobial efficacy of the stored coatings against two major bacterial pathogens, *Staphylococcus aureus* and *Staphylococcus epidermidis*, commonly found in chronic wounds. We did so both qualitatively via live/dead staining, as well as quantitatively via (2-methoxy-4-nitro-5-sulfophenyl)-5-[(phenylamino) carbonyl]-2H-tetrazolium (XTT) viability assay for up to 15 weeks in 5 weeks increments. Taken all together, we demonstrate that samples stored under FTV conditions retain the highest antimicrobial activity after 15 weeks and that this finding correlates with the retained concentration of nitroxides.

## 1. Introduction

A wound is described as chronic if it has not healed after eight weeks [1]. In Germany, approximately 1% of the population suffer from chronic wounds, and the global figure is 2.21% [2]. Because of our ageing population, this trend is rising [3]. For example, the likelihood to develop a chronic wound doubles between the ages of 50–60 and quadruples by the age of 80 [3]. As a result, treatment costs are estimated to account for 1–3% of the total health expenditure in developed countries [4,5,6]. Moreover, chronic wounds are a major contributor to mortality. For example, the five-year mortality rate of neuropathic and ischemic ulcers is around 50% [7]; which is higher than many forms of cancer. This high mortality from chronic wounds stems in large part due to the persistent infection that chronic wounds harbour. Because of this chronic infection by microorganisms, chronic wounds can’t progress in healing [8,9]. The most common pathogens found in chronic wounds are Gram-positive bacteria, namely S.aureus, S.epidermis, Enterococcusfaecalis and coagulase negative staphylococci [1]. A major problem is that these bacteria form biofilm that shields them from the immune system [10]. Biofilm diminishes the therapeutic window of antibiotics and provides an ideal environment for the exchange of genetic information between bacteria [11]. Because of these factors, antibiotic-resistant bacteria are common in chronic wounds; further reducing the treatment options of chronic wounds, which generally respond equally poorly to oral and topical antibiotics [11,12,13]. Unfortunately, the treatment options of chronic wounds have not changed significantly in the past century. Bandaging to control moisture levels, washing with saline and debridement are standard practice. Modern alternatives include hydrogel dressings and injectable hydrogels [14,15], hydrocolloid dressings, application of antiseptic moisturizing cream [16], dressings containing silver, alginates and foam dressings [17,18]. With relatively minor variations of these basic options, there is no conclusive research on which combination is the best. Additional therapies, such as hyperbaric oxygen-, ultrasound-, electromagnetic-, and vacuum-assisted therapy are currently being investigated as novel approaches [19,20]. However, there is no clear evidence for their benefit and they require specialised majy that restricts their use to hospitals [21].

**Figure 1:**
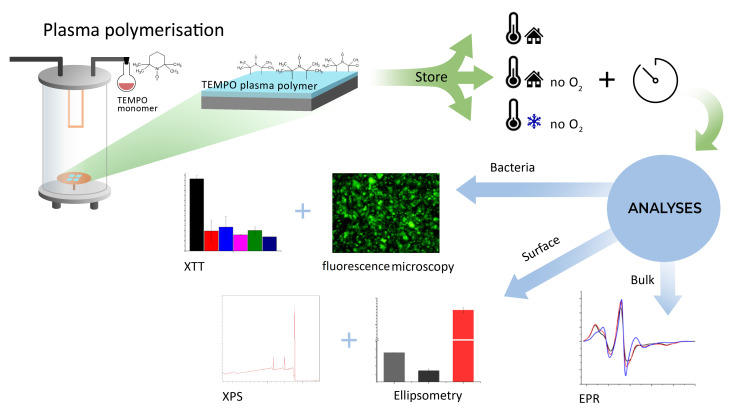
The aim of this work was as follows: The monomer TEMPO was polymerised to a plasma polymer using a plasma reactor. The resulting samples were stored under three different conditions: Room temperature, room temperature & vacuum and freezer temperature & vacuum. Samples were taken at different times and analysed via fluorescence microscopy, XTT assay, XPS, ellipsometry and EPR.

In contrast, we have recently demonstrated a viable alternative to treat chronic wounds via thin film coatings that release the anti-oxidant & long-lived nitroxide radical (2,2,6,6-Tetramethylpiperidin-1-yl)oxyl (TEMPO). These coatings were manufactured via the industrially scalable process [22,23,24,25] of plasma polymerisation (TEMPOpp) and slowly dissolve in aqueous environment, whilst being non-toxic [9,26,27]. The released antioxidants then act on the bacteria’s ability to form biofilm and, hence, the ability to colonise a wound. Thus, we aim with this novel method to upgrade commonplace bandages already in use for treating chronic wounds from “passive” passengers to “active” actors in the wound healing process. To the best of our knowledge, this method is unique because it can be applied to a broad range of existing wound dressing. However, the transition from in-vitro to in-vivo testing requires an appropriately long shelf-life of the thin films. Thus, by examining the shelf-life in-vitro first allows us to determine the time window under which the in-vivo testing needs to take place at a later stage. Thus, this work’s aim was twofold: First, we investigated how well TEMPOpp coatings store over time under three different conditions. These three conditions represent different types of accelerated ageing, according to the FDA’s recommendation on establishing shelf-life [28]. We learn from nitroxide mediating polymerisation (NMP), where the interaction of temperature and oxygen during the decomposition of nitroxides has already been investigated in detail [29,30]. Thus, we hypothesised that reducing the surrounding oxygen and lowering the temperature may slow down the nitroxides’ decay within TEMPOpp. Thus, we produced consistent batches of TEMPOpp that were then stored either at room temperature and air (RT), at room temperature and vacuum sealed (RTV) as well as freezer temperature (−18 ${}^{\circ }$C) and vacuum sealed (FTV). We then analysed the ageing of these coatings via the surface analysis with XPS and EPR. Second, we wanted to investigate how the three storage conditions affect the bacterium S.epidermis, and the other major chronic wound pathogen S.aureus [1]. The process is illustrated in Figure 1. From our results, we could see that indeed the storage conditions had a major impact, both on the surface chemistry and the biological activity.

## 2. Materials and Methods

### 2.1. Chemicals

All chemicals were used as received and according to manufacturer’s protocol. In addition, all biological growth media and solutions were autoclaved prior to use. Acetone (C${}_{3}$H${}_{6}$O) (Sigma-Aldrich, Macquarie Park, NSW, Australia), ammonium hydroxide (NH${}_{4}$OH) (Sigma-Aldrich, Macquarie Park, NSW, Australia), Ethanol (EtOH) (Sigma-Aldrich, Macquarie Park, NSW, Australia), hydrogen peroxide (H${}_{2}$O${}_{2}$) (Sigma-Aldrich, Macquarie Park, NSW, Australia), Hexamethyldisiloxane (HMDSO) (Sigma-Aldrich, Macquarie Park, NSW, Australia), LIVE/DEAD BacLight Bacterial Viability Kit L7007 (Thermo Fisher Scientific, Waltham, MA, USA), liquid nitrogen (LN${}_{2}$) (BOC, North Ryde, NSW, Australia), MilliQ water (UniSA, Adelaide, SA, Australia), phosphate buffered saline (PBS) (Astral Scientific, Taren Point, NSW, Australia), TEMPO (Sigma-Aldrich, Macquarie Park, NSW, Australia), tryptic soy broth (TSB) (Sigma-Aldrich, Macquarie, Park NSW, Australia), Virkon (Alltech Lienert, Roseworthy, SA, Australia), XTT (Roche, Basel, Switzerland).

### 2.2. Consumables

Silicon wafers 100 mm, Czochralski method (CZ), [100] 5–10 ohm, P/boron, 350 ± 25 obtained from MMRC Australia.

### 2.3. Database and Software

Endnote X9.3.3 was used to manage the literature. ESR Studio 2.0 from Freiberg Instruments, Freiberg, Germany was used to measure the EPR. Gen5 and KC4 from BioTek, Winooski, USA were used to measure the absorbance with the microplate readers. To create all graphs OriginPro 8.5.0 from OriginLab, Northampton, MA, USA was used. As software for ellipsometry WVASE32 Ver. 3.770 from J.A. Woollan, Lincoln, NE, USA was used. XPS data were processed and analysed with CasaXPS 2.3.16 Casa Software Ltd, Teignmouth, United Kingdom.

### 2.4. Plasma Reactor

**Figure 2:**
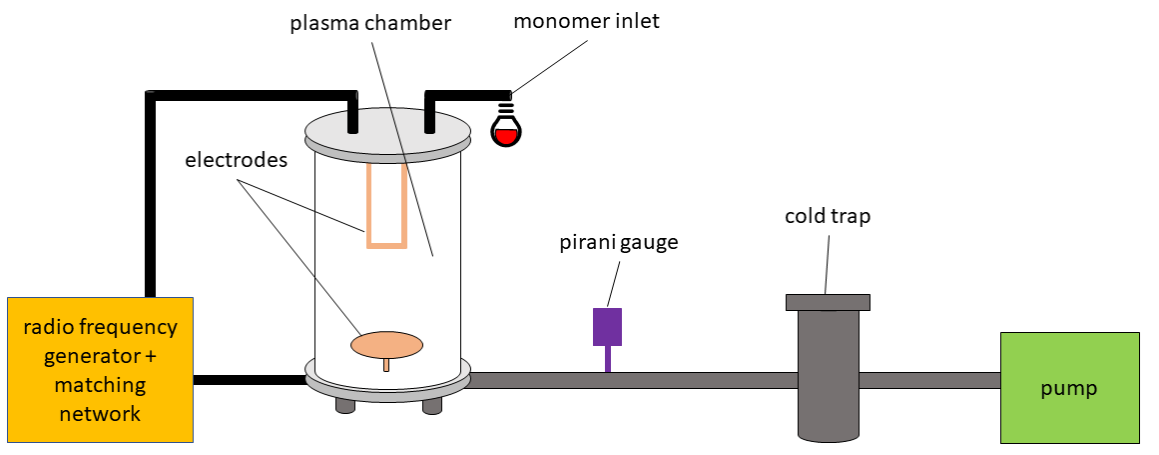
The layout plasma reactor’s layout; incuding the position of the RF generator, electrodes, monomer inlet, pirani gauge, cold trap and pump.

The chamber has a diameter of 16.5 cm. At a height of 4.5 cm the plate electrode with a diameter of 12 cm is mounted. The outer wall of the chamber is made of glass and measures 34 cm in height. From the ceiling of the chamber there are two connected electrodes with a width of 8 cm. 12 cm are between the rod electrodes and the plate electrode. There are two connections on the chamber. One connection for the monomer. The other connection is for connecting the chamber to a RF generator. At the bottom of the chamber there is a connection to ground the chamber. Figure 2 shows the schematic structure of the plasma reactor. The pump creates a vacuum in the direction of cold trap. The cold trap is filled with liquid nitrogen, so that particles and substances that come into contact with it freeze there and thus do not contaminate the pump downstream. The air inlet serves to break the vacuum. The pirani gauge is used to measure the pressure in the chamber. This reactor has already been used to produce TEMPO coatings under different conditions [9,26,27].

### 2.5. Standardised Instruments

A densitometer DEN-1 from the company Grant-bio was used to determine the McFarland value. A digital control heated water bath with in-built shaking tray from Thermoline Scientific was used to incubate the bacteria. The Foodsafer Vacuum sealer was used to prepare the sample for exclusion of oxygen. A Nikon digital sight DS-L3 at a 490 nm excitation wavelength and live-dead optical filters were used to take the fluorescence microscopy images. To measure the absorbance at 490 nm an ELx800 absorbance microplate reader from Bio-Tek with the corresponding software Gen5 was used. To measure the absorbance at 540 nm a Synergy HT multi-detection microplate reader from Bio-Tek with the appropriate software KC4 was used.

### 2.6. Bacteria

S.aureus strain ATCC 13301 and S.epidermis strain ATCC 35984 were used. One nutrient agar plate was streak plated with S.aureus and one with S.epidermis and placed in the incubator for 24 h at 37 ${}^{\circ }$C. A bacterial colony was taken and filled into a 50 mL Cellstar tube together with 20 mL TSB and placed in the incubator for 24 h at 37 ${}^{\circ }$C. The suspension was adjusted to McFarland of 1 (1 × 10${}^{8}$ CFU/mL) and then further diluted to a final concentration of 1 × 10${}^{6}$ colony forming units (CFU)/mL in TSB. The dilutions were verified via plating out. 600 μL of the suspension was pipetted on the samples and incubated for 24 h at 37 ${}^{\circ }$C.

### 2.7. Sample Preparation

#### 2.7.1. Cutting Silicon Wafers

Silicon wafer were cut into 1 cm × 1 cm on a Disco DAD321 Dicing Saw.

#### 2.7.2. RCA Cleaning

Pre cut silicon wafer were heated together with a solution of ammonium hydroxide, hydrogen peroxide and MilliQ water in a ratio of 1:1:5 to 70 ${}^{\circ }$C for 30 min. Then washed twice with sufficient MilliQ water. Afterwards the silicon wafers were covered in EtOH and sonicated for 5 min. The same procedure was repeated with acetone. As last step the silicon wafers were stored in fresh acetone [31].

#### 2.7.3. Plasma Polymer Deposition

In order to produce coatings using the plasma generator, the first step was to fill the cold trap with liquid nitrogen. Furthermore, the chamber was pumped down to base pressure <20 mTorr. Following, a 50 mL round bottom flask filled with the monomer was attached to the chamber. After that the monomer was degassed by cooling the glass flask with liquid nitrogen and opening the valve to draw the gas into the chamber. The flask was then reheated and the valve closed. This process was repeated three times. Then the respective substrate was placed on the plate electrode. To open the chamber, it must first be vented. After the respective substrate was placed in the chamber, the chamber was pumped back down to base pressure. Both silicon wafers and NaCl were coated, the silicon wafers for the antimicrobial testing and the NaCl for the EPR. The conditions were set according to Table 1.

#### 2.7.4. Sample Preservation for Storage

The samples were placed into 24-wellplates (RT), additionally vacuum packed (RTV) or vacuum packed and placed into an industrial grade freezer at −18 ${}^{\circ }$C (FTV). To avoid condensation onto the samples which may cause additional ageing effects, only vacuum-packed samples were placed into the freezer.

#### 2.7.5. Ellipsometry

A J.A. Woollam V-vase ellipsometer was used to verify the thickness of the coatings produced in the plasma generator. The measurement was carried out at a wavelength of 400–1000 nm, in 10 nm steps for the angles 65${}^{\circ }$, 70${}^{\circ }$ and 75${}^{\circ }$. A two-layer Cauchy model was loaded to optimize the optical parameters and minimising the squared error. Thus the calculated thickness was obtained via the Cauchy-Euler equation [32]:

$$a_{n}x^{n}y^{(}{}^{n}{}^{)}\left(x\right)+a_{n}{}_{-}{}_{1}x^{n}{}^{-}{}^{1}y^{(}{}^{n}{}^{-}{}^{1}{}^{)}\left(x\right)+...+a_{0}y\left(x\right)=0.$$

#### 2.7.6. X-ray Photoelectron Spectroscopy

For XPS a Kratos Axis Ultra DLD spectrometer was used. A monochromatic Al Kα X-ray source operating at 225 W, corresponding to 1486.6 eV and the raster size of 0.3 × 0.7 mm was used as source. To neutralise the build-up charge an electron flood gun was used. The survey spectra were measured at 160 eV pass energy with 0.5 eV steps with a dwell time of 55 ms. For high-resolution the C 1s spectra were collected at 20 eV pass energy with 0.1 eV steps. To analyse the data a Shirley baseline correction was used. The C-C peak in the C 1s spectra were adjusted to 284.8 eV in order to calibrate all spectra.

#### 2.7.7. Electron Paramagnetic Resonance

In order to measure the EPR spectra a Magnettech MiniScope MS 5000 with the respective software EPR Studio 2.0 was used. The coated NaCl mentioned in Section 2.7.3 was inserted into a quartz EPR tube with a diameter of 3.6 mm. Different settings were used: 336 ± 5 mT, power 10 mW, modulation frequency 100 kHz, amplitude 1 × 1000, sweep time one minute. For kinetic measurements a measuring point was set every five minutes. The geometry of the EPR device does not allow the use of silicon wafers as substrate for analysis. Thus, instead we opted for sodium chloride (NaCl) as a substrate for plasma polymerisation. The TEMPOpp coated NaCl crystals are not active in EPR and are easily filled into the machine. The coated NaCl TEMPOpp samples were produced under identical conditions and stored in identical fashion as the TEMPOpp on silicon wafers for RT, RTV and FTV.

#### 2.7.8. Fluorescence Microscopy

The bacteria were treated as described in Section 2.6. After removing the supernatants from the samples, each sample was washed twice with 500 μL deionised water. Each sample was covered with a Live/Dead-stain (LIVE/DEAD BacLight Bacterial Viability Kit L7007) and incubated for 20 min at 37 ${}^{\circ }$C. Subsequently the samples were washed twice with 500 μL deionized water and placed onto a microscope slide. It is important that the samples do not dry out because otherwise the bacteria die and thus falsify the result. Within ten minutes the corresponding pictures were taken with a microscope.

#### 2.7.9. XTT Assay

The mixing ratio was as follows, XTT Labeling Reagent 1.5 mL and Electron-coupling Reagent 30 μL. The bacteria were treated as described in Section 2.6. After that the suspension was removed and the samples were washed twice with 600 μL PBS. PBS + XTT was placed into an empty well for the negative control. 300 μL XTT were added to each sample and then incubated for five hours at 37 ${}^{\circ }$C. 100 μL of each sample was transferred into a 96-well plate and measured in a well plate reader at 490 nm. Afterwards a one-way ANOVA with Dunnett’s multiple comparison test was performed to check the significance of the individual measuring points within the groups.

## 3. Results and Discussion

We have previously demonstrated that TEMPO can be plasma polymerised into TEMPOpp and these coatings’ ability to act on biological systems; such as the quorum sensing of bacteria [9,26,27]. Due to the radical nature of TEMPO, slow decay is inevitable. Thus our question was, whether we can extend the TEMPOpp coatings’ shelf life via storage conditions? Two major factors affecting radical decay are the presence of oxygen and storage temperature [29,30]. After the coatings’ production, we verified their thickness via ellipsometry. Subsequently, the three storage conditions applied were RT, RTV and FTV. To evaluate the coatings’ chemistry, we conducted XPS and EPR analysis after 15 weeks of storage. We tested the coatings in 5-week intervals to correlate surface chemistry and biology, starting at time point 0. It has already been demonstrated that TEMPOpp has an antimicrobial effect for S.epidermis [9] but it has not yet been tested for another major pathogen in chronic wounds, S.aureus [1]. Thus we evaluated the antimicrobial effect quantitatively via fluorescence microscopy and quantitatively via XTT assay for both pathogens.

### 3.1. Thickness Measurement of Plasma Polymers

For this work, three different plasma polymers were necessary. First, plasma polymerised Ethanol (EtOHpp), because it is suitable as a control sample by providing good adhesion for both S.aureus and S.epidermis [33]. Second, plasma polymerised Hexamethyldisiloxane (HMDSOpp) was used as an adhesion layer for the TEMPOpp coatings to prevent delamination from the silicon substrate [34,35]. Third, the TEMPOpp plasma polymer that was deposited on top of the HMDSOpp. One should note that the TEMPOpp thickness consists of the total thickness of HMDSOpp and TEMPOpp together. The respective deposition conditions can be seen in Table 1.

**Figure 3:**
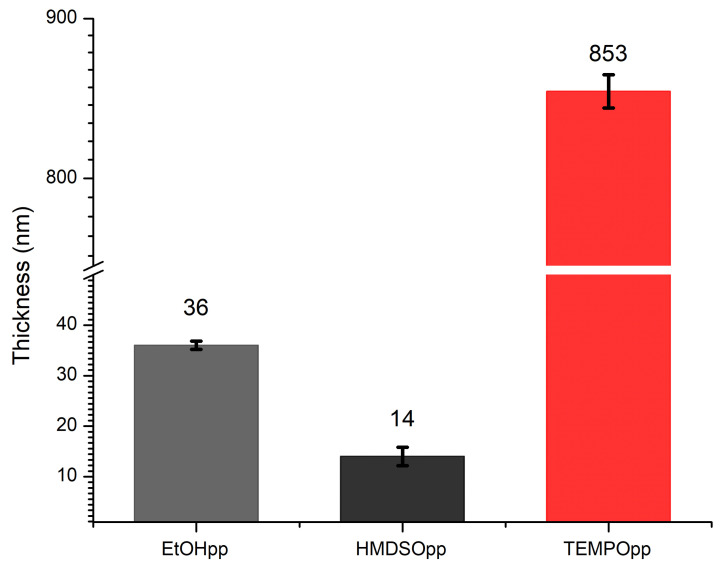
Thickness of EtOHpp, HMDSOpp and TEMPOpp on silicon wafer, error bars are the respective standard deviation of the population.

We chose ellipsometry as a simple method to verify the quality of the resulting plasma polymers. The results can be seen in Figure 3. Our reasoning was that if all parameters in the manufacturing process remain the same, i.e., pressure, temperature, monomer, flow rate, power and reactor architecture, the layers per batch have the same thickness; a well-known quality of plasma polymerisation [36,37].

Previous reports for the thickness of EtOHpp under the same conditions ranged from 30–40 nm [33], which is within the range what has been achieved in this work. The same applies for the HMDSOpp adhesion layer [34]. Our previous work on TEMPOpp obtained an average layer thickness of 850 nm [9], which is very close to our average thickness of 853 nm. In view of the above, all three plasma polymers are within the expected range of previously published literature, and hence ellipsometry has been proven a useful quality control tool.

### 3.2. XPS

**Figure 4:**
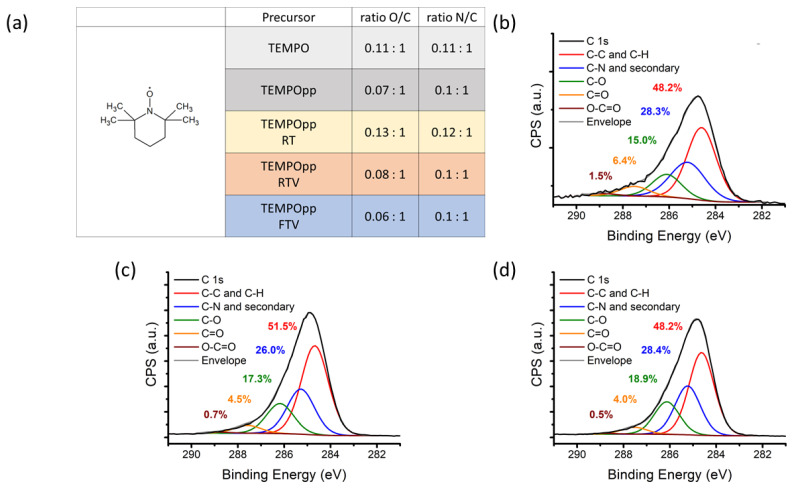
(**a**) Atomic composition of TEMPOpp RT, RTV and FTV. High resolution C 1s spectrum of TEMPOpp stored at RT (**b**), RTV (**c**) and FTV (**d**) for 15 weeks.

The XPS analysis result are seen in Figure 4. All samples were 15 weeks old when measured, and for comparison, the theoretical values of TEMPO were also included. Further, it was decided to compare the oxygen to carbon and nitrogen to carbon ratios. Firstly, the results reflect our previous research, both for the O/C & N/C ratio as well as C 1s. Thus, demonstrating that the plasma polymerisation of TEMPO was a success and can be repeated reliably and homogenously, as demonstrated in our previous works [9,26,27]. Secondly, the N/C ratio was almost identical for fresh, RT, RTV and FTV samples. This finding is in contrast to the O/C ratio. Here we find that RT samples had the highest ratio, followed by RTV, fresh and finally FTV, with the lowest ratio. This is an interesting finding as it implies that the coatings aren’t losing volatile nitrogen compounds over time, regardless of storage conditions. This implies that the remaining free radicals in the plasma polymer react with oxygen from air to form peroxy and hydroxyl radicals [38]. The effect was most pronounced for RT samples and less so for RTV ones. This can be explained by the residual oxygen already contained in the package that could interact with the plasma polymer. The effect of oxygen reacting with polymers and changing the surface’s composition over time is known [39]. For example, studies have shown that polymers stored in air for 360 days and then analysed using XPS show an increase in oxygen content [39]. Furthermore, we assume that the FTV samples had the lowest O/C ratio content because due to the lower oxidation speed at lower temperatures [40].

When comparing the high-resolution recording C 1s of Figure 4b RT, Figure 4c RTV and Figure 4d FTV, there were only marginal differences. The ratio of components was similar among the samples, and hence it wasn’t clearly evident which chemical species differed between the three storage conditions. However, one can observe a slight shift from the lower oxidation stages of carbon to the higher ones; this applies mostly for the (O-C=O) and (C=O) which mirror the increased O/C ratio.

### 3.3. EPR

**Figure 5:**
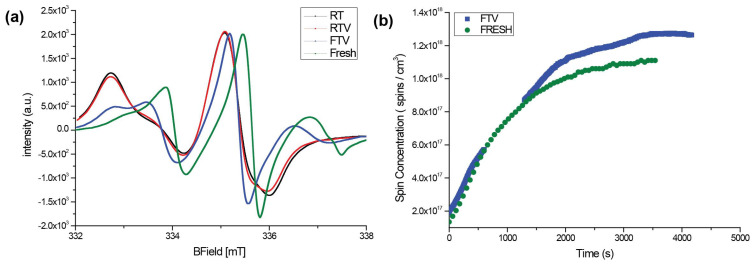
(**a**) EPR spectra of TEMPOpp RT, RTV and FTV on NaCl after 10 weeks (**b**) Time evolution of spin concentration upon exposure to air of 15-week-old TEMPOpp FTV vs. fresh TEMPOpp.

A more specific method to detect nitroxides is EPR, which is highly sensitive to unpaired electrons [41,42]. Figure 5a reveals the ageing behaviour of TEMPOpp. The minima & maxima of FTV are shifted the least from fresh TEMPOpp. In comparison, the EPR signal of RT and RTV are shifted almost identically. This shift can be attributed to the formation of hydroxyl radicals and the interaction between TEMPO and oxygen has been investigated in other studies [43,44,45]. Admittedly, these studies relate to TEMPO and not TEMPOpp, but the EPR signal obtained suggests that analogue conclusions can be drawn. Furthermore, this oxidation effect was also observed with other nitroxides [46]. Thus, as one can see, even FTV has undergone some degree of oxidation which lies somewhere between fresh TEMPOpp and either RT & RTV. Lastly, the nitroxide decay suggests re-freezing does not reverse the ageing process: Once refrigeration and vacuum are interrupted, the samples commence to irreversibly age.

Another point of interest was to compare the spin concentration of fresh TEMPOpp and TEMPOpp FTV samples after they are unsealed. This indicates whether the coatings after storing under ideal conditions behave closely to freshly produced ones. For this purpose, a kinetic measurement of freshly produced TEMPOpp and a 15-week old FTV sample was measured (Figure 5b). As observed previously [27], there is an increase of radical density over time. Furthermore, the radical intensity among the two TEMPOpp samples, fresh and FTV, behaves similarly. This result suggests that TEMPOpp FTV stored samples after 15 weeks aligns closely to freshly produced TEMPOpp.

### 3.4. Microbiological Testing

Two major pathogens found in chronic wounds were chosen for the microbiological testing [10]. On the one hand, S.epidermis is commonly found on the skin, is known to form a good biofilm [47] and has been investigated in context with TEMPOpp before [9]. On the other hand, S.aureus, as it can be a highly problematic pathogen to treat [48].

#### 3.4.1. Fluorescence Microscopy

**Figure 6:**
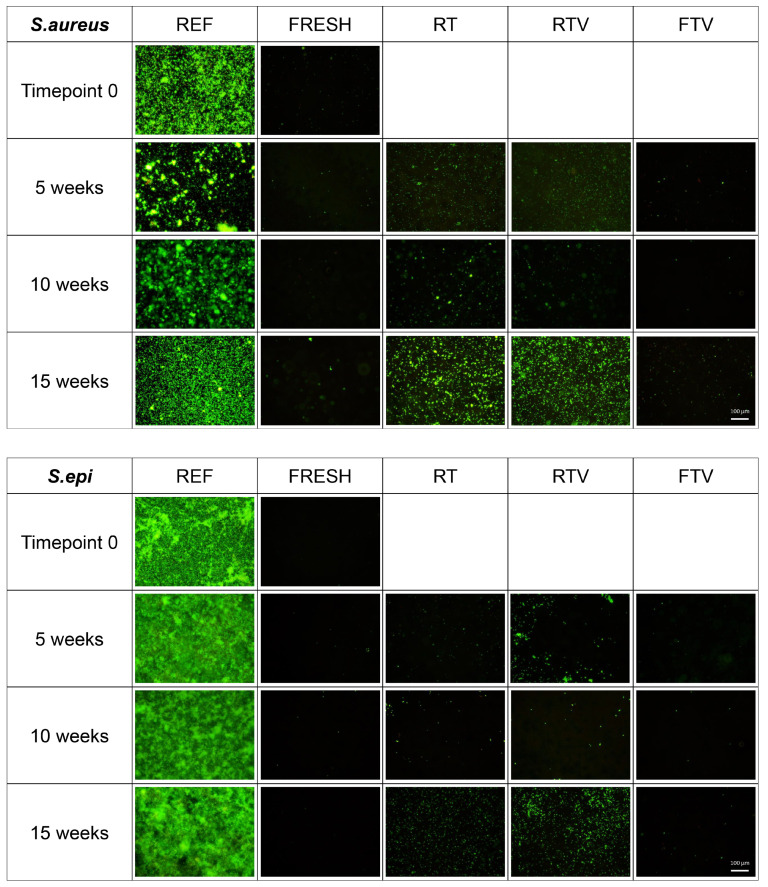
Fluorescence microscopy coatings after incubation with S.aureus (**top**) and S.epidermis (**bottom**) for 24 h, *n* = 2, qualitative live/dead images (20×).

We used fluorescent live/dead staining [49] to qualitatively evaluate the differences among the samples. The results are seen in Figure 6 for S.aureus (top) and S.epidermis (bottom). EtOHpp was used as control, and it is apparent that S.aureus does form a biofilm on the control; albeit with some variation, which is a known observation in microbiology [50]. In contrast, S.epidermis formed a biofilm on each control; which it is known for [47]. Fresh TEMPOpp has a clearly visible effect both on S.aureus and S.epidermis for each measurement as no biofilm was evident. This is a reassuring result as it demonstrates the reproducible potency of fresh TEMPOpp to prevent biofilm formation by both types of bacteria. By comparison, the three storage conditions elicit different efficiencies. TEMPOpp stored under RT and RTV result in similar amounts of bacterial growth at each of the three timepoints and for both types of bacteria. As storage time increases, in increments of 5 weeks, it becomes evident that the RT and RTV storage conditions lead to a loss in activity. This is more pronounced in the case of S.aureus than S.epidermis; particularly after 15 weeks. A possible reason for this observation could be the difference in quorum sensing between S.aureus and S.epidermis, i.e., the communication by chemical signals through which bacteria measure the cell population’s density. In contrast, TEMPOpp stored at FTV had a similar performance as fresh TEMPOpp [51]. This visual result mirrors the presence of nitroxides and a lower amount of hydroxyl decay products; as discussed in Section 3.3.

#### 3.4.2. XTT Assay

**Figure 7:**
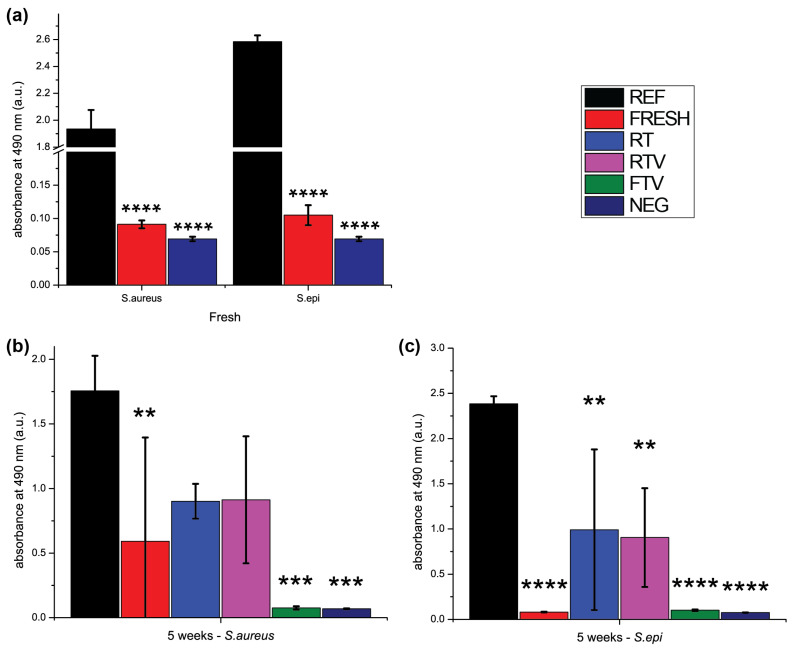

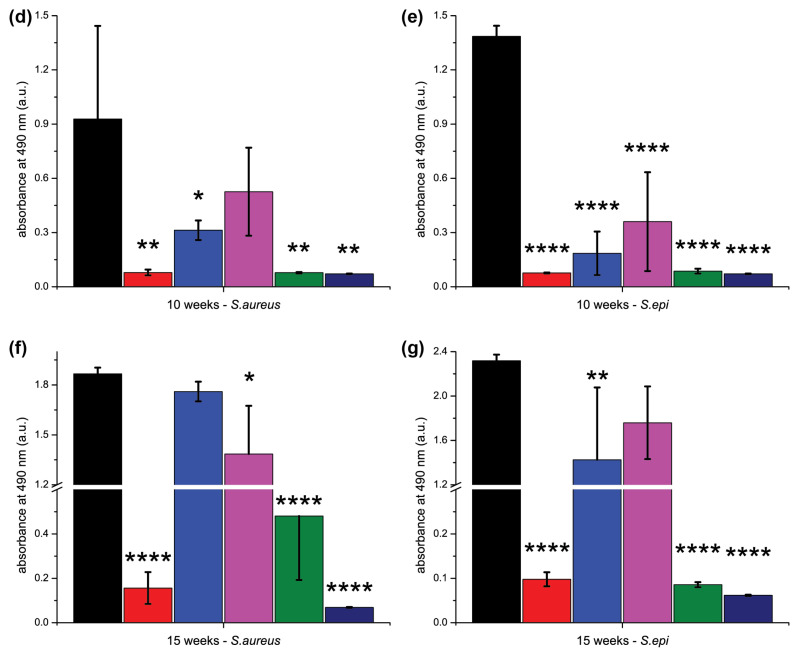
XTT results of the TEMPOpp coatings after incubation with either S.aureus or S.epidermis for 24 h. (**a**) Fresh samples (**b**,**c**) after 5 weeks (**d**,**e**) after 10 weeks (**f**,**g**) after 15 weeks. *n* = 4, error bars are the respective standard deviation of the population, statistical significance was determined using a one-way ANOVA with Dunnett’s multiple comparison test, * denotes significant difference compared to control EtOH (*p* < 0.05), * *p* < 0.05, ** *p* < 0.01, *** *p* < 0.001 and **** *p* < 0.0001.

To complement the qualitative microbiological results qualitatively, we conducted the XTT assay in parallel. XTT is a relative quantitative test that indirectly measures the bacteria’s metabolic activity. As seen in Figure 7, fresh TEMPOpp is highly capable of suppressing the metabolic activity of both S.aureus and S.epidermis [52]. One should note that the metabolic activity of this particular strain of S.aureus appeared to be always lower than that of the S.epidermis strain. After five weeks of storage under the three different conditions, changes in microbiological efficacy became evident. For both bacteria, the XTT results mirrored the ones from fluorescent microscopy. Namely that both RT and RTV had higher growth than fresh TEMPOpp. Fresh TEMPOpp for S.aureus was slightly elevated because one of the quadruplicates had growth on it. It remains a question whether this was likely due to faulty coatings or other reasons. We consciously decided to include all quadruplicates, to also evaluate and showcase the coatings’ reproducibility. Considering that this was the only outlier among all fresh TEMPOpp (for both types of bacteria as well as for fluorescent microscopy and XTT assay), we are satisfied with the reproducibility for laboratory purposes (see the Supplementary Materials for the complete data list). However, it has become evident that more work needs to be done for up-scaling to achieve even fewer potentially faulty samples. Overall, the results demonstrate that FTV was highly active. Interestingly, the negative control and FTV were nearly identical; further highlighting its potency. This trend of antimicrobial activity (Fresh = FTV < RT = RTV) is also seen in the later time points 10 and 15 weeks. The only time when TEMPOpp FTV appears to start losing potency is in the case of S.aureus after 15 weeks. This may be a true biological correlation to the EPR results where we did see minor oxidation occurring in FTV. In comparison, both TEMPOpp RT and RTV had nearly identical metabolic activity as the reference for both types of bacteria; mirroring the EPR results once again.

## 4. Conclusions

Chronic wounds remain an unsolved healthcare problem affecting a sizeable portion of the population. The methods used currently treating chronic wounds are either outdated or not easily applicable outside a hospital setting. In contrast, we have demonstrated that TEMPOpp thin film coatings can tackle the infections within chronic wounds. Thus, TEMPOpp could be used to transform any wound dressing from “passive” passenger to “proactive” actor in the healing process. To evaluate if these coatings could be used in a medical setting, more information on TEMPOpp coatings’ shelf-life was needed; as they contain the long-lived nitroxide radical TEMPO. We did exactly this in this work, where we hypothesised that both temperature and air exclusion could prolong the coating’s activity window. For this reason, the samples were manufactured identically and split into three lots: RT, RTV and FTV. We demonstrated that the chemical changes over time were only marginally noticeable in the atomic composition and interconnection, as determined via XPS after 15 weeks. A lower oxygen to carbon ratio was evident for the samples stored under freezer temperatures and air exclusion. However, only minor changes in the C1s spectra could be seen among all three storage conditions, mainly for the (O-C=O) and (C=O) components. In comparison, EPR showed a distinct difference in the quality of radicals after 15 weeks. Whereas RT and RTV shifted markedly towards hydroxyl radical decay, FTV still retained the most nitroxide radicals. Furthermore, upon exposing FTV to air, the radical density behaved closely to that of “fresh” TEMPOpp samples. These observations mirrored the results in the qualitative and quantitative microbiological testing closely. Namely, we could demonstrate for the first time that another major pathogen in chronic wounds, S.aureus, is inhibited by TEMPOpp coatings. S.epidermis is also reliably inhibited, and both types of bacteria react similarly to the three different storage conditions. Namely, RT and RTV start losing efficacy within 5 weeks, whereas FTV is active against both bacteria up to 15 weeks; albeit slightly less so for S.aureus. This was seen clearly from the lack of biofilm formation (live/dead fluorescent microscopy) and suppressed metabolism (XTT). These encouraging results are an important stepping stone to demonstrate that the coatings’ shelf life is sufficient for medical applications and is comparable to the shelf life of some vaccines and medications which are stored cold [53,54]. Our next steps will be first to test the coatings’ efficacy after even more prolonged time points under FTV conditions and use even lower storage temperatures, as well storage under inert gases (Nitrogen or Argon); the latter used routinely in the packaging industry. And second, take these coatings from *in-vitro* to *in-vivo* testing to evaluate their performance in a realistic animal chronic wound model. Both of these next goals are essential before these coatings can be assessed in a clinical trial.

## Figures and Tables

**Table 1:** Plasma conditions of HMDSO, EtOH and TEMPO plasma polymer.

Characteristic	Monomer	Power (W)	Pressure (mTorr)	Time (min)
adhesion layer	HMDSO	25	154	1
cleaning	air	50	200–400	1
surface A	EtOH	40	100	5
surface B	TEMPO	10	104	30

## Supplementary Materials

The following are available online at https://www.mdpi.com/article/10.3390/antibiotics10040362/s1, Table S1: fresh TEMPOpp XTT S.aureus, Table S2: 5 weeks old TEMPOpp XTT S.aureus, Table S3: 10 weeks old TEMPOpp XTT S.aureus, Table S4: 15 weeks old TEMPOpp XTT S.aureus, Table S5: fresh TEMPOpp XTT S.epidermis, Table S6: 5 weeks old TEMPOpp XTT S.epidermis, Table S7: 10 weeks old TEMPOpp XTT S.epidermis, Table S8: 15 weeks old TEMPOpp XTT S.epidermis.
