# Supplementary material for: Shelf-Life Optimisation of Plasma Polymerised (2,2,6,6-Tetramethylpiperidin-1-yl)oxyl (TEMPOpp) Coatings; A New Possible Approach to Tackle Infections in Chronic Wounds
Source: Antibiotics (Basel). 2021 Mar 29;10(4):362. doi: 10.3390/antibiotics10040362 (PMC8067075; doi:10.3390/antibiotics10040362)
Supplement: Supplementary file 1 [file antibiotics-10-00362-s001.pdf]

Supplementary Material

Shelf-Life Optimisation of Plasma Polymerised (2,2,6,6-Tetramethylpiperidin-1-Yl)Oxyl (Tempopp) Coatings; A New Possible Approach to Tackle Infections in Chronic Wounds

Kilian Böttle1,‡, Krasimir Vasilev1and Thomas Danny Michl1,∗,‡

Kilian Böttle, Dr. Thomas Michl, Prof. Krasimir Vasilev
School of Engineering, University of South Australia, Mawson Lakes, SA 5095, Australia
Email: kili.boettle@gmail.com

The values in the following tables (S1-S8) are absorbance values measured at 490 nm.

**time point 0**	**XTT**	**S.aureus**				
	EtOH	TEMPO new	TEMPO RT	TEMPO RTV	TEMPO FTV	neg
1	1.783	0.099	-	-	-	0.065
2	1.808	0.089	-	-	-	0.073
3	2.076	0.094	-	-	-	0.072
4	2.076	0.083	-	-	-	0.067
average	1.93575	0.09125				0.06925
stdv	0.14052824	0.00593191				0.0033448

Table S1: fresh TEMPOpp XTT S. aureus.

**time point 1**	**XTT**	**S.aureus**				
	EtOH	TEMPO new	TEMPO RT	TEMPO RTV	TEMPO FTV	neg
1	1.717	1.979	0.967	0.546	0.085	0.065
2	1.912	0.229	0.672	0.338	0.083	0.074
3	1.337	0.082	1.019	1.212	0.082	0.071
4	2.06	0.076	0.948	1.555	0.054	0.066
average	1.7565	0.5915	0.9015	0.91275	0.076	0.069
stdv	0.271031825	0.803413499	0.1350268	0.49164996	0.01274755	0.003674235

Table S2: 5 weeks old TEMPOpp XTT S. aureus.

**time point 2**	**XTT**	**S.aureus**				
	EtOH	TEMPO new	TEMPO RT	TEMPO RTV	TEMPO FTV	neg
1	1.487	0.077	0.085	0.11	0.076	0.068

2	1.345	0.079	0.369	0.461	0.109	0.073
3	1.36	0.075	0.07	0.763	0.082	0.072
4	1.35	0.073	0.217	0.107	0.078	0.072
average	1.3855	0.076	0.18525	0.36025	0.08625	0.07125
stdv	0.058849384	0.002236068	0.1205246	0.27345875	0.01331118	0.001920286

Table S3: 10 weeks old TEMPOpp XTT S. aureus.

**time point 3**	**XTT**	**S.aureus**				
	EtOH	TEMPO new	TEMPO RT	TEMPO RTV	TEMPO FTV	neg
1	1.931	0.139	1.689	1.692	0.441	0.066
2	1.849	0.124	1.841	1.169	0.472	0.069
3	1.851	0.086	1.722	1.649	0.099	0.07
4	1.836	0.276	1.79	1.03	0.91	0.07
average	1.86675	0.15625	1.7605	1.385	0.4805	0.06875
stdv	0.037539146	0.071785705	0.0590445	0.2900974	0.28794314	0.00163936

Table S4: 15 weeks old TEMPOpp XTT S. aureus.

**time point 0**	**XTT**	**S.epi**				
	EtOH	TEMPO new	TEMPO RT	TEMPO RTV	TEMPO FTV	neg
1	2.546	0.081	-	-	-	0.065
2	2.606	0.118	-	-	-	0.073
3	2.649	0.104	-	-	-	0.072
4	2.532	0.117	-	-	-	0.067
average	2.58325	0.105				0.06925
stdv	0.04704984	0.01491643				0.0033448

Table S5: fresh TEMPOpp XTT S. epidermis.

**time point 1**	**XTT**	**S.epi**				
	EtOH	TEMPO new	TEMPO RT	TEMPO RTV	TEMPO FTV	neg
1	2.249	0.087	0.075	0.115	0.108	0.073
2	2.456	0.078	1.886	1.528	0.094	0.076
3	2.374	0.078	1.872	0.7	0.091	0.074
4	2.455	0.081	0.133	1.276	0.11	0.078
average	2.3835	0.081	0.9915	0.90475	0.10075	0.07525
stdv	0.084482247	0.003674235	0.8877507	0.54586966	0.0083479	0.001920286

Table S6: 5 weeks old TEMPOpp XTT S. epidermis.

**time point 2**	**XTT**	**S.epi**				
	EtOH	TEMPO new	TEMPO RT	TEMPO RTV	TEMPO FTV	neg
1	1.487	0.077	0.085	0.11	0.076	0.068
2	1.345	0.079	0.369	0.461	0.109	0.073
3	1.36	0.075	0.07	0.763	0.082	0.072
4	1.35	0.073	0.217	0.107	0.078	0.072
average	1.3855	0.076	0.18525	0.36025	0.08625	0.07125
stdv	0.058849384	0.002236068	0.1205246	0.27345875	0.01331118	0.001920286

Table S7: 10 weeks old TEMPOpp XTT S. epidermis.

**time point 3**	**XTT**	**S.epi**				
	EtOH	TEMPO new	TEMPO RT	TEMPO RTV	TEMPO FTV	neg
1	2.413	0.085	1.76	1.873	0.086	0.059
2	2.295	0.089	1.817	2.004	0.081	0.062
3	2.28	0.125	0.299	1.962	0.081	0.063
4	2.279	0.092	1.829	1.199	0.095	0.062
average	2.31675	0.09775	1.42625	1.7595	0.08575	0.0615
stdv	0.055930202	0.01592757	0.6513399	0.32704319	0.0057173	0.0015

Table S8: 15 weeks old TEMPOpp XTT S. epidermis.
